# In Vivo Voltammetric Imaging of Metal Nanoparticle-Catalyzed Single-Cell Electron Transfer by Fermi Level-Responsive Graphene

**DOI:** 10.34133/research.0145

**Published:** 2023-05-22

**Authors:** Qing Xia, Rui Liu, Xueqin Chen, Zixuan Chen, Jun-Jie Zhu

**Affiliations:** ^1^ State Key Laboratory of Analytical Chemistry for Life Science, School of Chemistry and Chemical Engineering, Nanjing University, Nanjing 210023, P. R. China.; ^2^ Shenzhen Research Institute of Nanjing University, Shenzhen 518000, P. R. China.

## Abstract

Metal nanomaterials can facilitate microbial extracellular electron transfer (EET) in the electrochemically active biofilm. However, the role of nanomaterials/bacteria interaction in this process is still unclear. Here, we reported the single-cell voltammetric imaging of *Shewanella oneidensis* MR-1 at the single-cell level to elucidate the metal-enhanced EET mechanism in vivo by the Fermi level-responsive graphene electrode. Quantified oxidation currents of ~20 fA were observed from single native cells and gold nanoparticle (AuNP)-coated cells in linear sweep voltammetry analysis. On the contrary, the oxidation potential was reduced by up to 100 mV after AuNP modification. It revealed the mechanism of AuNP-catalyzed direct EET decreasing the oxidation barrier between the outer membrane cytochromes and the electrode. Our method offered a promising strategy to understand the nanomaterials/bacteria interaction and guide the rational construction of EET-related microbial fuel cells.

## Introduction

Electrochemically active bacteria (EAB) can transfer electrons from the metabolism of organic sources to solid electron receptors [1–4]. Such extracellular electron transfer (EET) process plays an important role in the biogeochemical cycle of elements, and it is widely involved in energy technologies [5–8] such as microbial fuel cells (MFCs), microbial electrosynthesis, and analyte detection [9,10]. Current efforts primarily aimed at the enhancement of the electron transfer kinetics between EAB and electrodes [11–14], and considerable progress has been made in improving the performance of MFCs by optimizing the microorganism selection [15–17] and battery construction [18,19]. Among them, metal/metallic oxide nanomaterials were reported to demonstrate the capability of facilitating either the direct or indirect EET at the microorganism/electrode interface via the interaction with cytochromes [4,20–22]. Knowledge of how nanomaterials enhance the electron transfer rate at the microorganism/electrode interface will help the development of high-performance MFCs. However, the detailed mechanism is still unclear. One most popular view is that the good conductivity of nanomaterials could provide a route to connect bacteria with others [4]. Another hypothesis attributed it to the catalytical properties of metal nanomaterials [23]. However, it has not been confirmed by experimental results because the complexity of biofilm hinders the understanding of detailed interaction between the electrode and bacteria [24,25].

Single-cell analysis eliminated the limitation of the microorganism and provides a powerful method for promoting the investigation of the EET mechanism [18,26–37]. For example, single-cell EET was usually studied by detecting the single-cell current output with micro-/nanoelectrodes [26,27,29,31]. The Lieber group measured the output current from individual *Shewanella oneidensis* MR-1 (*S. oneidensis* MR-1) [26] and *Geobacter sulfurreducens* DL-1 [29] by combining nanoelectrodes and brightfield microscopy. The Nakanishi group used optical tweezers to capture a single *S. oneidensis* MR-1 cell on a microelectrode for electrochemical current measurement [27]. These micro/nano-electrode-based methods provided the highly sensitive and quantitative current measurements of single cells at around 100 fA. However, the rare outer membrane cytochromes (Omc) hindered the voltammetric study of nanomaterial-enhanced EET mechanism at the single-cell level. In our previous work, Fermi level-responsive graphene electrode (FGE) was proposed to offer an extreme detection limit at the attoampere level that enabled the observation of single-molecular level electron transfer in cytochromes [38]. Thus, it has the potential capabilities to study the EET mechanism at the single-cell level.

In this work, the direct EET in single MR-1 cells was studied using FGE. The transparent single-layer graphene (SLG) allows for the transmission of scattering from single cells, which could be collected by the objective. The FGE-based single-cell voltammetric imaging could reduce the possible effects of secreted mediators and allow the electrical characterization of cytochromes or their metal nanoparticle complexes without forming a biofilm. Unlike the metal-enhanced EET mechanism based on the increment in the overall catalytical current, the single and subcellular results revealed a nanomaterial-catalyzed EET mechanism. AuNPs can catalyze the electron transfer of single MR-1 cells by reducing the oxidation potential of Omc, thus improving the EET performance. It provided a potential platform for high-throughput and rapid screening of electricity-producing bacterial cells and constructing MFCs.

## Results

**Fig. 1.**
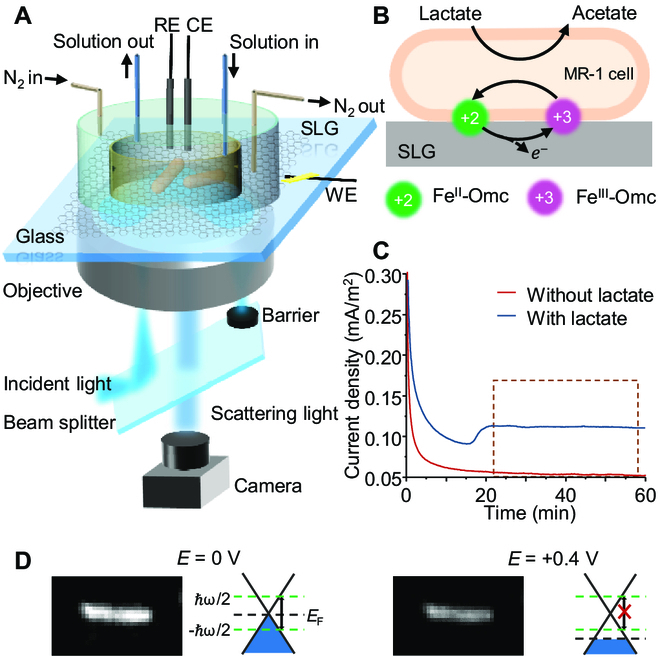
Schematic illustration of principle of FGE. (A) Schematic illustration of the construction of the optical setup for the FGE. WE, working electrode; RE, reference electrode; CE, counter electrode. (B) Schematic diagram of Omc-based direct extracellular electron transfer process of MR-1. (C) Chronoamperometry of monodispersed MR-1 cells in the presence of 18 mM lactate on the SLG electrode surface at +0.4 V (versus Ag/AgCl). The stable plateau marked with a dashed box indicated the sustained current output from MR-1. (D) Scattering image of MR-1 under different potentials and the corresponding energy diagrams of SLG under the cell.

The setup for FGE was demonstrated in Fig. 1A. SLG was transferred onto a cover slide. It acted as the transparent working electrode (WE) with an Ag/AgCl reference and a platinum counter electrode. The electrolyte was M9 buffer. All potentials in this work were relative to the reference electrode. To attain anaerobic condition and allow the solution exchange, we constructed a sealed incubator on an SLG substrate as described in the previous work [39]. Two concentric polydimethylsiloxane (PDMS) wells are attached and sealed to the substrate, allowing for solution exchange and N_2_ atmosphere. To eliminate the interference from the flavin-mediated indirect EET process, we continuously renew the electrolyte solutions during the chronoamperometric experiments to remove flavins secreted by MR-1 (see Materials and Methods) that would induce the negative shift in the oxidation potential (Fig. S1) [40,41]. To image the scattering light from single cells, collimated white light was directed onto the cover slide via a high numerical aperture oil immersion objective to generate the evanescent field, which was further scattered by the cells. A barrier was placed behind the objective to stop the reflected light, and only the scattering light was directed to a camera to form dark-field images.

During the direct electron transfer pathway, MR-1 was thought to mediate EET to the SLG electrode through abundant Omc molecules, such as MtrC and OmcA (Fig. 1B). Chronoamperometry was used to initially detect the electricity-generating current of monodispersed MR-1 cells in the presence of 18 mM lactate on the SLG electrode surface at +0.4 V (versus Ag/AgCl) [11,29]. Although the current output was weak due to the rare cells (~10^12^ cells/m^2^; see Materials and Methods) on the electrode, a stable plateau corresponding to sustained current output was observed after 20 min (Fig. 1C, blue curve). In contrast, the current recorded from a control system without lactate was flat and with no current output (Fig. 1C, red curve). The activated cells during the stable plateau period were chosen for further studies. In our previous work, FGE was described to have the capability of converting the redox state of cytochrome *c* to the change in Rayleigh scattering [38]. Similar results were observed in the MR-1 cells. As shown in Fig. 1D, the quinone pool in the inner membrane could reduce the Omc at the zero potential [42], which led to relatively strong scattering of the cells. When *E* increased to 0.4 V, which was higher than the redox potential of Omc on the SLG, the scattering started to be forbidden due to the decrease of the graphene’s Fermi level (*E*_F_).

**Fig. 2.**
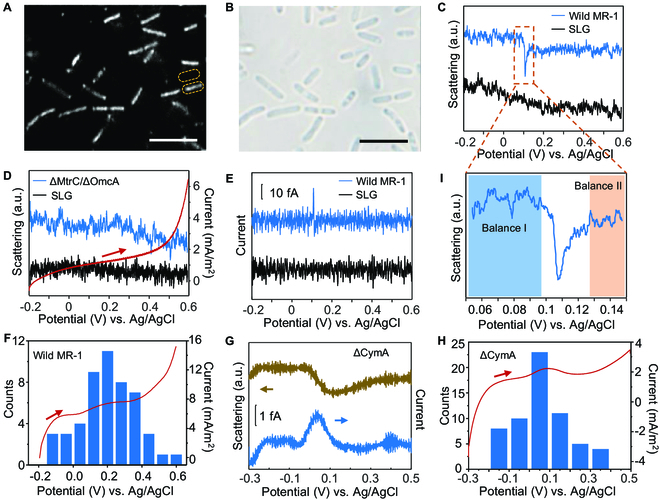
Single-cell voltammetry of MR-1 cells. (A and B) Scattering (A) and transmitted (B) image of single MR-1 cells on the SLG. Scale bar, 5 μm. (C) Scattering intensities of SLG (black) and the MR-1 cell (blue) labeled with yellow dotted lines in (A) during the linear scanning of applied potential. (D) Scattering intensities of SLG (black) and the ΔMtrC/ΔOmcA mutant cell (blue) during the linear scanning of applied potential. Linear sweep voltammogram of ΔMtrC/ΔOmcA mutant cells measured with a potentiostat (red). (E) Converted linear sweep voltammograms of SLG (black) and MR-1 cell (blue) calculated from the scattering intensity in (C). (F) Histograms showing the distribution of single oxidation events (*n* = 50). The whole electrode's linear sweep voltammogram (red line) was recorded with a potentiostat. (G) Scattering light intensity of a ΔCymA mutant cell during the linear scanning of applied potential. (H) Histograms showing the distribution of the oxidation potentials of single ΔCymA mutant cells (*n* = 60). The bulk linear sweep voltammogram (red line) was synchronously measured with a potentiostat. (I) Magnification of the region in (C) marked with the dashed box.

The strong redox state-dependent scattering offered a tool for understanding the electron transfer performance of single MR-1 cells. Figure 2A shows the clear, rod-shaped scattering profiles of the mono-dispersed MR-1 cells, fitting the transmitted image very well (Fig. 2B). Linear sweep voltammetry (LSV) scans were performed on the graphene electrode to study the redox behavior of Omc at the single-cell level. When the applied potential was scanned linearly from −0.2 to +0.6 V with a scan rate of 0.01 V s^−1^, the rod-shaped scattering of a single MR-1 cell (labeled with dashed box) had a sudden drop in the scattering intensity at ~0.1 V (Fig. 2C, blue curve), while the neighboring graphene area only showed a slow scattering intensity drift (Fig. 2C, black curve). More examples were demonstrated in Fig. S2 and Video S1. Please note that the sensitivity of SLG to the surface charges was smaller than the multilayer graphene that showed apparent potential dependent scattering changes because of the weak scattering [38]. However, it did not affect the performance of brighter objectives on SLG (Fig. S3). Accordingly, sudden drop in the scattering was attributed to the rapid oxidation of rich Omc molecules. To confirm it, the MR-1 mutant lacking genes encoding Omc (ΔMtrC/ΔOmcA) was used as a control that only demonstrated the similar scattering intensity drift to the graphene area in response to the applied voltage. The LSV curve recorded with a potentiostat also showed no oxidation peaks (Fig. 2D). According to our previous work, the increasing charge density (*n*) changed linearly with the scattering intensity (Δ*I/I*) at the interface [38]. Through the corresponding optical-electrochemical conversion model, the scattering signal was processed in time and space, and the local charge density change on a single cell could be obtained. When the electron transfer reaction occurred on SLG without any diffusion, the current density *i* of a single cell could be simply measured by the following equation:

$$i=\mathrm{Ae}\frac{d\left(∆I/I\right)}{\mathrm{dt}}$$ (1)

where *e* is the elementary charge, ∆*I*/*I* is the relative scattering change of a single cell, and *A* is a constant that measures the response slope of ∆*I*/*I* to the charge density of graphene [38].

Equation 1 allowed for the conversion from the scattering intensity to the local voltammograms of the cell (for details, see Materials and Methods). Figure 2E displays the converted LSV curves of the graphene area and the cell in Fig. 2A. A spike-like oxidation peak of ~25 fA was observed in the cell area at around 0.1 V, while the graphene region only showed a background noise of ~5 fA. The oxidation peak potentials of LSV curves were measured in many single MR-1 cells (Fig. S4). As shown in Fig. 2F, histograms of these oxidation peaks showed concentrated distribution near 0.2 V. Dramatically, the LSV curve of the whole electrode recorded with a potentiostat also displayed an oxidation peak at this potential (Fig. 2F, red curve). The good correlation revealed that the apparent oxidation event was the statistical result of single oxidation events of Omc in MR-1 cells. We noted that the oxidation potential is more positive than the reported results [41,43]. We attributed this to 2 main reasons. The first reason is the electrode material that can affect the redox potentials. Another influence factor might be the environment of Omcs. Pure proteins could have better contact with the electrode that usually decreases the overpotential. The peak current *I*_peak_ of single oxidation events was also related to the EET performance of MR-1 cells. As shown in Fig. S5, the higher scattering intensities of MR-1 cells, which indicate the more Omc on cell membrane, the higher peak currents were observed. Figure S6 featured the distribution of *I*_peak_ of abundant single MR-1 cells, under both turnover and nonturnover conditions. Under nonturnover conditions in the absence of substrate lactate, *I*_peak_ was found to be concentrated at around 10 fA, which was attributed to the oxidation of Omc with the limited EET. On the contrary, in the turnover process, MR-1 cells metabolized lactate and transfer electrons to the Omc. As a result, more Omc molecules would be oxidized during the LSV scans and the statistical mean *I*_peak_ increased to ~20 fA. Such an increment of *I*_peak_ in the turnover process was in good agreement with the variation in the output current (Fig. 1C). It was worth noting that the extraordinary sharp oxidation peak could not be explained by the Nernst behavior of the oxidation state of redox compounds, which should result in broader oxidation and reduction peaks. Therefore, we attribute the sharp oxidation peak to the fast electron transfer kinetics in Omc once the potential was scanned over the oxidation potential. We noted that similar fast-decaying peaks in the single-cell charge transport measurement were observed with microelectrodes, which is attributed to the quick discharge of the accumulated electrons in cell membrane [29]. It could be regarded as the quick oxidation of cytochromes. Thus, the sharp oxidation peak was attributed to a “broken-and-rebuilding” process of the balance between the internal electron transfer (IET) and the electrical oxidation of Omc during the EET process. Under anaerobic conditions, the redox cycling of quinone in MR-1 cells metabolized the lactate. It transported electrons through the Mtr pathway to the outer membrane, which was further accepted by the high-potential electrode. IET would likely reduce the Omc, while the external electrode tended to oxidize it. Thus, the apparent oxidation rate of Omc *r* was expressed, *r* = *r*_O_ − *r*_R_, where *r*_R_ is the reduction rate of Omc induced by IET and *r*_O_ is the electrical oxidation rate.

Before we explained the observation of sharp oxidation peaks, we needed to figure out the pure electrical oxidation peaks of the Omc without the contribution of EET. Thus, a control experiment was carried out with an MR-1 mutant lacking genes encoding CymA (ΔCymA). CymA is a type of cytochromes located in the inner membrane and acts as a mediator to transfer electrons from the quinone pool to the Omc. Thus, IET in ΔCymA mutant was intercepted. The ΔCymA mutant was initially cultured under strict anaerobic condition, and the Omc was reduced by flavins or other electron shutters. During the electrochemical experiments, we continuously renew the electrolyte solutions to remove these electron shutters. As a result, the Omc became isolated species, and only the electrical oxidation rate *r*_O_ was considered. As shown in Fig. 2G, unlike MR-1 cells, a slight decrease appeared in the scattering intensity of a single ΔCymA mutant cell at ~0.05 V when the applied potential was scanned from −0.3 to 0.5 V (brown curve). More examples were shown in Fig. S7. The calculated LSV curve displayed a broad oxidation peak with a half-height width of ~100 mV, which was reconciled with the Nernst thermodynamics (blue curve). Furthermore, the statistical oxidation peak potentials of more than 30 cells showed a concentrated distribution at ~0.05 V, much smaller than that of wild MR-1 cells (~0.2 V), in good agreement with the LSV curve synchronously recorded by the commercial potentiostat (Fig. 2H).

Accordingly, the cause of the sharp oxidation peaks of MR-1 cells is distinct. Figure 2I shows details of the sudden decrease in the scattering intensity of the marked region in Fig. 2C. One can observe an asymmetric fall-and-rise signal at ~0.1 V. Initially, most Omc were in the reduction state when the potential was scanned from −0.2 V. In consideration of the fact that only oxidized Omc were required to serve as a conduit to mediate electron transfer from metabolic pathways to the solid electrode [27], there was a balance between *r*_R_ and *r*_O_, while both of them were small at negative potentials. When the potential was scanned positively, *r*_O_ started to increase linearly at small overpotential, which could still be equalized by the increasing *r*_R_. Thus, there was no change in the scattering intensity during this process even if the potential was scanned over the peak potential (Balance I). As the potential was scanned toward more positive potential, the efficiency of oxidation increased exponentially, breaking the balance between *r*_O_ and *r*_R_, and Omc started to be oxidized. At the same time, electrons are transported from CymA through the Mtr pathway to the SLG electrode, linking the EET at the outer membrane to cation pumping and thus the membrane potential across the inner membrane [28]. The membrane hyperpolarization led to the quick discharge of the accumulated electrons in the outer membrane [29]. As a result, the charge-sensitive scattering showed a fast-decaying peak at ~0.105 V followed by a lower stable plateau where Omc were completely oxidized (Balance II). The whole balance–broken–rebuilding process lasted ~30 mV. In the new balance, the MR-1 cell had more oxidized Omc than in Balance I that promoted the EET.

**Fig. 3.**
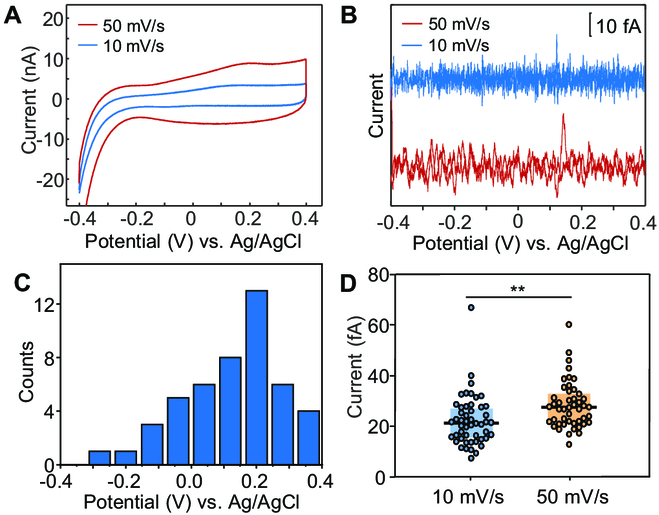
Scan rate-dependent voltammetry of MR-1 cells. (A) Cycle voltammograms of MR-1 cells on the SLG at the scan rate of 10 (blue) and 50 mV/s (red) recorded by the potentiostat. (B) Converted cycle voltammograms of MR-1 cells on the SLG at the scan rate of 10 (blue) and 50 mV/s (red) recorded by the potentiostat. (C) Histograms showing the distribution of single oxidation events of MR-1 cells at the scan rate of 50 mV/s (*n* = 47). (D) Oxidation peak current of MR-1 at the scan rate of 10 (*n* = 50) and 50 mV/s (*n* = 47). Line, median; bottom and top of boxes, first and third quartiles, respectively. ** (*P* ≤ 0.01) denoted statistically significant difference.

We further investigated the influence of the scan rate on the single-cell oxidation peaks. Figure 3A displays the cyclic voltammetric (CV) curves of the whole SLG electrode incubated with MR-1 cells at different scan rates. Both 2 CVs only had an oxidation peak, and the oxidation peak currents showed a strong dependence with scan rate, confirming the immobilization of the Omc on the electrode [44]. The converted single-cell CV curves demonstrated similar results. As shown in Fig. 3B, only the oxidation peaks were observed at both scan rates. Although the peak potential at 50 mV/s was slightly more positive than that at 10 mV/s, the concentrated peak potential distribution of 47 cell samples near ~0.2 V was close to that at the scan rate of 10 mV/s (Figs. 2F and 3C). In addition to that, the peak current at 50 mV/s was higher than at 10 mV/s. The scan rate-dependent peak current was confirmed by comparing the statistic results at each scan rate (Fig. 3D). The good correlation revealed that the apparent oxidation event was the statistical result of single oxidation events of Omc in MR-1 cells.

Metal nanoparticles were reported to be capable of facilitating the direct EET process of microbial cells via the interaction with Omc, leading to marked enhancement in the MFC’s performance [4,45]. However, the interaction mechanism of metal nanoparticles/bacteria had not been well understood. One most popular view was that the excellent conductivity of metal nanomaterials provided a route to connect bacteria with others or the electrode that primarily contributes to the EET enhancement. It could explain the output current increment; however, whether the interaction affected the electron transfer activity of Omc was still unclear. Aiming that, we studied the interaction mechanism of metal nanoparticles/bacteria at the single-cell level. Gold nanoparticles (AuNPs) are a classic noble nanomaterial that not only can promote electron transfer but also have good biocompatibility [46–49].

**Fig. 4.**
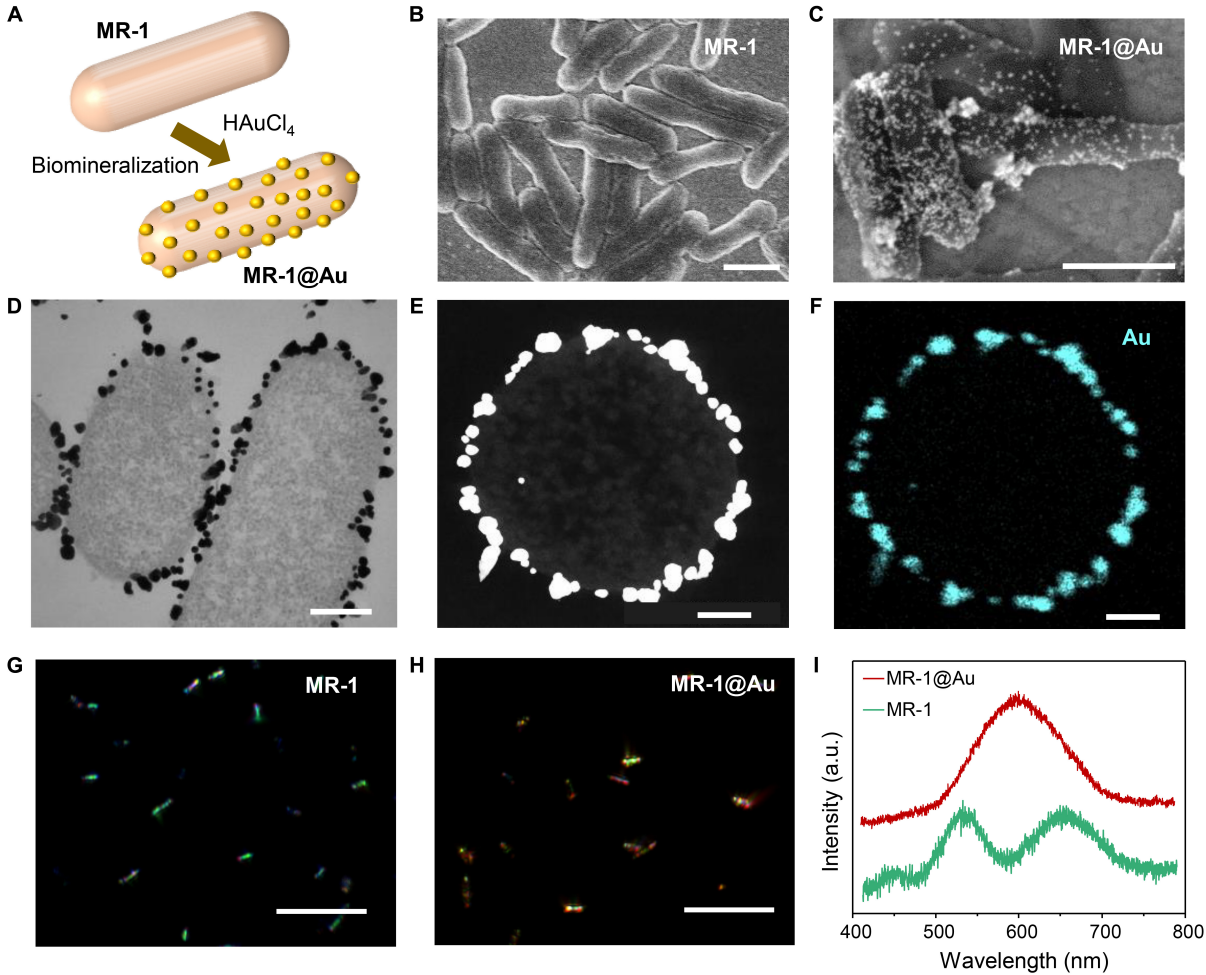
Biomineralization of the MR-1 cells. (A) Schematic illustration of the formation of MR-1@Au cells. (B and C) SEM images of (B) MR-1 and (C) MR-1@Au cells. Scale bar, 1 μm. (D) TEM image of MR-1@Au cells. Scale bar, 200 nm. (E and F) High-angle annular dark-field scanning transmission electron microscopy (HAADF-STEM) image (E) and element mapping image (F) of the cross-section slice of an MR-1@Au cell. Scale bar, 100 nm. (G and H) Scattering images of (G) MR-1 and (H) MR-1@Au cells. Scale bar, 10 μm. (I) Scattering spectra of a single MR-1 (green) and MR-1@Au cell (red).

AuNP-modified MR-1 (MR-1@Au) cells were taken as an example to evaluate the effect of noble metal modification on the EET capacity of single MR-1 cells (Fig. 4A). Scanning electron microscopy (SEM) images demonstrated that MR-1@Au cells had uniform AuNPs on the surface (Fig. 4B and C). Further insights into the distribution of AuNPs were obtained by the TEM and element mapping of the cross-section slice of an MR-1@Au cell (Fig. 4D to F). Strikingly, abundant AuNPs exist only on the surface of the outer membrane. Figure 4G and H displays the dark-field images of MR-1 and MR-1@Au cells, respectively, and both the cells were monodispersed. The MR-1 cells showed uniform green scattering (Fig. 4G) that was attributed to the intrinsic structural color scattering under the anaerobic condition (Fig. 4I, green curve) [39]. In remarkable contrast, the aggregates of AuNPs induced a plasmonic resonance scattering at 600 nm (Fig. 4I, red curve), resulting in the orange scattering of MR-1@Au cells (Fig. 4H). To probe the effect of AuNP modification on the viability of MR-1, a LIVE/DEAD BacLight bacterial viability kit was used to differentiate live cells (green fluorescence) from dead cells (red fluorescence). MR-1@Au and native MR-1 displayed strong green and slight red fluorescence (Fig. S9), indicating that biomineralization caused no damage to the cell activity. We also studied the electrochemical behavior of MR-1@Au. Similar to native cells, the current output of MR-1@Au was also observed during the chronoamperometry analysis (Fig. S10).

**Fig. 5.**
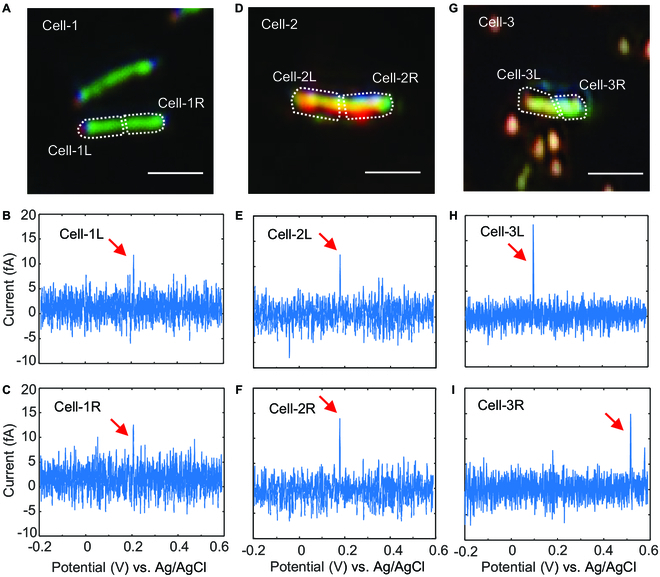
Subcellular voltammetry of the asymmetric electron transfer activities in MR-1 cells. (A) Scattering image of an MR-1 cell labeled with Cell-1. Scale bar, 1 μm. Converted linear sweep voltammograms of the MR-1 cell at locations marked with (B) Cell-1L and (C) Cell-1R. (D) Scattering image of an MR-1@Au cell labeled with Cell-2. Scale bar, 1 μm. Converted linear sweep voltammograms of the MR-1 cell at locations marked with (E) Cell-2L and (F) Cell-2R. (G) Scattering image of an MR-1@Au cell labeled with Cell-3. Scale bar, 1 μm. Converted linear sweep voltammograms of the MR-1 cell at locations marked with (H) Cell-3L and (I) Cell-3R.

Further insight into the EET ability of MR-1@Au cells could be obtained by comparing the LSV curves with the bare MR-1 cells. The classic LSV curves of 2 types of cells, recorded with the potentiostat, demonstrated similar shapes (Fig. S11). However, the single-cell LSV curves highlighted something differently. Figure 5A shows a bare MR-1 cell (Cell-1) scattering image that demonstrated uniform bright green structural color scattering from left to right except for 2 curved tips [39]. During the potential scanning, both the left part (marked with Cell-1L) and the right part (marked with Cell-1R) had an oxidation peak at ~0.2 V (Fig. 5B and C). Similar results were found in the AuNP-modified MR-1 cell (Cell-2) that showed uniform bright orange scattering (Fig. 5D). Two oxidation peaks at ~0.2 V were observed on both sides (Cell-2L and Cell-2R), which was more negative than in Cell-1 (Fig. 5E and F). It was worth noting that the oxidation peaks of 2 sides of one cell were always kept at the same potentials, regardless of the AuNP modification or not (more examples in Fig. S12). However, things became different when the 2 sides of an MR-1 cell were modified with varying amounts of AuNPs. As shown in Fig. 5G, the orange color dominated on the left side of Cell-3 (Cell-3L), while the right side (Cell-3R) still showed the green scattering. That is to say, the left side contained more AuNPs. The asymmetric AuNP modification induced multiple oxidation peaks in the LSV curve (Fig. S13). The peak at 0.1 V was contributed by the left side (Fig. 5H), and the peak at 0.5 V came from the right side (Fig. 5I). Oxidation peaks at 2 different potentials were attributed to the nonuniform density of AuNPs at the surface of the cell. AuNPs can facilitate the electron transfer between Omc and graphene that induced a negative shift in the oxidation potential. Thus, the left part (Cell-3L) modified with more AuNPs demonstrated higher EET ability than the right part (Cell-3R).

**Fig. 6.**
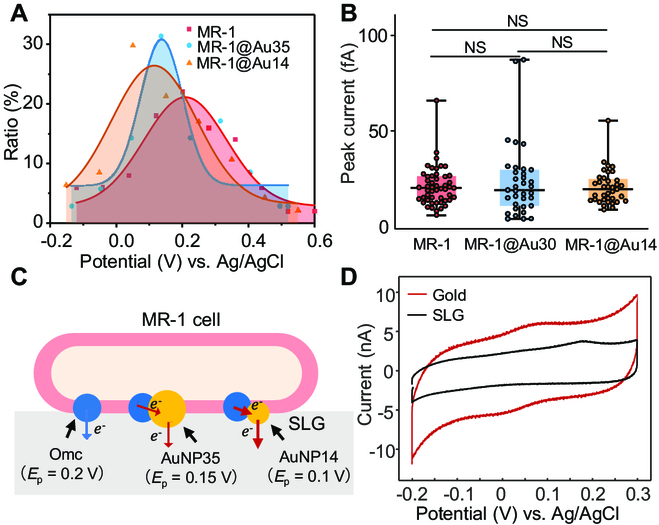
Metal-catalyzed electron transfer in single MR-1 cells. (A) Fitted potential distribution curves of the single oxidation events of MR-1 (red, *n* = 50), MR-1@Au35 (blue, *n* = 35), and MR-1@Au14 (orange, *n* = 37). Scatters were experimental values (red, MR-1; blue, MR-1@Au35; orange, MR-1@Au14). (B) Oxidation peak current of MR-1 (*n* = 50), MR-1@Au35 (*n* = 35), and MR-1@Au14 (*n* = 37). Line, median; bottom and top of boxes, first and third quartiles, respectively; bottom and top of colored lines, minimum and maximum of data, respectively. NS (*P* ≥ 0.05) denoted no statistically significant difference. (C) Schematic illustration of catalyzed direct EET by AuNPs in a single MR-1 cell. *E*_p_ was the oxidation potential of Omc. Blue and red arrows indicated slow and fast electron transfer processes, respectively. (D) Cycle voltammograms of MR-1 cells on the SLG (black) and gold (red) electrodes were recorded by the potentiostat. Scan rate is 0.01 V s^−1^.

The peak shift induced by AuNPs was further confirmed with the statistical results from plenty of single-cell samples. As shown in Fig. S8, we prepared 2 types of MR-1@Au cells. MR-1@Au35 have uniform AuNPs with a central size of ~35 nm, while the size of AuNPs on MR-1@Au14 was much smaller (~14 nm). Both the sizes of AuNPs were similar to the Omcs (~10 nm) [50]. Figure 6A displays the distribution of oxidation peak potentials of 3 types of cells, including bare MR-1 (red), MR-1@Au35 (blue), and MR-1@Au14 (orange). Bare cells showed a broad distribution of oxidation peak potentials from 0 to 0.4 V with a central potential at ~0.2 V. After being modified by 35-nm AuNPs, the distribution became more concentrated to lower potentials (0 to 0.3 V) that induced the central potential shifting to 0.15 V. The central peak potential of MR-1 modified by 14-nm AuNPs was further shifted to ~0.1 V. To eliminate the contribution from the Omc amount deviations among these cells, the peak current *I*_peak_ was also investigated during the LSV scanning (Fig. 6B). Dramatically, the 3 types of MR-1 cells demonstrated similar statistical mean *I*_peak_ of ~20 fA, suggesting the similar amount of Omc near the electrode. *I*_peak_ of MR-1@Au cells was also investigated in the nonturnover process without lactate (Fig. S14). Although a similar distribution of oxidation potentials was demonstrated, the statistical mean *I*_peak_ of MR-1@Au cells decreased from 20 fA to 10 fA, similar to the MR-1 cells (Fig. S6). The lacking lactate inhibited the EET, leading to less reduced Omc than in the turnover process. As a result, the amount of oxidized Omc decreased during the LSV scanning, as well as the peak currents.

Accordingly, we proposed a hypothesized interaction mechanism of AuNPs and Omc that reduced the oxidation potential of MR-1 cells (Fig. 6C). MR-1 had a wide range of redox potential due to many reasons, such as the different coupling and interactions between the hemes in Omc and the electrode and the activity of Omc [51,52]. As described above, the oxidation potential of a single MR-1 cell was determined by both the intracellular (*r*_R_) and extracellular factors (*r*_O_). It was well known that noble metals give better electron transfer kinetics than graphene surfaces [53,54]. In the biomineralization of AuNPs, Omc transferred electrons to Au^III^ and transformed them into AuNPs. The in situ formed AuNPs acted as an “electrical nanoplug” for the alignment of Omc on the graphene and for the electrical wiring of the Fe^3+^/Fe^2+^ redox centers [55]. Such an electrical wiring established a more efficient electronic conduit between hemes and the electrode that facilitated the electron transfer from the Omc to the electrode. Thus, *r*_O_ increased, leading to the balance breaking at lower potentials. Smaller AuNPs demonstrated better electron transfer efficiency and therefore lower potentials than bigger AuNPs. However, the oxidation potential of MR-1 cells could not become lower than that of the pure Omc (Fig. 2I). Therefore, we observed a more concentrated distribution of oxidation peaks at lower potentials (Fig. 6A). We confirmed this hypothesis by comparing the CV curves of wild-type MR-1 on the gold and SLG electrode, respectively. As shown in Fig. 6D, the SLG electrode displayed the oxidation peaks at ~0.2 V. In remarkable contrast, the oxidation potential shifted to 0.05 V in the CV of the gold electrode. The gold electrode's lower oxidation potential proved that the AuNPs could reduce the oxidation potential of MR-1 cells by giving better electron transfer kinetics than graphene.

## Discussion

In summary, we developed a general method to investigate the EET capacity of single electricity-producing bacteria before and after noble metal modification. A better understanding of EET was the key to developing new EAB, determining the fundamental limitations of MFCs, and improving their power extraction. The challenge of studying the direct pathway of EET mainly came from the difficulty to exclude biofilm and secreted mediators in population-level experiments. The FGE-based single-cell analysis could reduce the possible effects of secreted mediators and allow the electrical characterization of cytochromes or their metal nanoparticle complexes without forming a biofilm. Unlike the reported mechanism where metal nanoparticles provided a route to connect bacteria with others in the biofilm, our results revealed a nanomaterial-catalyzed EET mechanism where AuNPs could reduce the oxidation potential of Omc, further improving the EET performance. In addition, FGE's high spatiotemporal resolution capability would enable it to detect the electron transfer in different parts of a single bacterial cell, proving that AuNPs can catalyze the direct EET by reducing the oxidation barrier between Omc and the electrode. The redox reactions at the bacteria/electrode interface were the driving force for output currents. As a result, a lower potential could facilitate the EET process and benefit the development of high-power density MFCs. Our method provided a potential platform for high-throughput and rapid screening of electricity-producing bacterial cells and constructing MFCs.

## Materials and Methods

### Chemicals

MR-1 was purchased from the American Type Culture Collection (Manassas, VA, USA). All mutants of MR-1 were kindly provided by Y.-C. Yong (Jiangsu University). Luria–Bertani (LB) broth was purchased from Sigma-Aldrich (Shanghai, China). Glutaraldehyde (2.5%) was diluted from 25% glutaraldehyde, purchased from Sinopharm Reagent (Beijing, China). HAuCl_4_ was obtained from Shanghai Reagent Company (Shanghai, China). Chemical vapor deposition (CVD) SLG on copper foil was purchased from Nanjing XFNANO Materials Tech Co. Ltd. Ultrapure water with a resistivity of 18.2 MΩ cm was produced using a Milli-Q apparatus (Millipore) and used in the preparation of all solutions. Cover slides were purchased from Thorlabs Co. Ltd. PDMS was prepared using Sylgard 184, Dow Corning. All media and solutions were sterilized before use.

### General techniques

SEM images were collected on a JEOL JSM-7800F scanning electron microscope (Hitachi Co., Japan). The electrochemical cell was placed on the 100× oil immersion objective (numerical aperture = 1.49) and equipped with a Nikon Ti-E inverted microscope for dark-field scattering imaging. A barrier was placed at the objective's back focal plane to stop the reflected light, and only the scattering light was directed to a charge coupled device (CCD) camera (AVT Pike F-032B). A broadband light source (EQ-99XFC LDLS, Energetiq Technology) was used for incident illumination. True-color dark-field images were captured with a color-cooled digital camera (DS-RI1, Nikon). Fluorescent imaging was also carried out on this microscope (Ti-E, Nikon, Japan), equipped with a mercury lamp (Nikon Intensilight C-HGFI). Electrochemical experiments were performed on a workstation (ACFBP1, PINE Research Instrumentation).

### Cell culture

MR-1 cells were cultured in a 50-ml centrifuge 15-ml LB broth medium at 30 °C overnight on a shaker (150 rpm). The bacterial cells were harvested by centrifugation (5,000 rpm, 5 min) and dispersed in sterile water or M9 buffer solution (22 mM KH_2_PO_4_, 42 mM Na_2_HPO_4_, 85.5 mM NaCl, and 1.0 mM MgSO_4_) for further use [11].

### SEM analysis

Cells were suspended in a 2.5% glutaraldehyde solution over 1 h for cell fixation and then dehydrated in increasing concentrations of ethanol solution (25%, 50%, 75%, 85%, 95%, and 100%). Finally, indium tin oxide glass was dipped into the ethanol solution of cells and dried under ambient conditions for SEM tests [11,48].

### Viability analysis

Cell viability was studied with the LIVE/DEAD BacLight bacterial viability kit. Cells were stained with the dye mixture (SYTO 9/propidium iodide, 1:1) in the dark for 20 min; fluorescence images of stained cells were obtained in the fluorescein isothiocyanate and tetramethyl rhodamine isothiocyanate mode, respectively [56].

### Biomineralization

MR-1 was cultured in LB broth with continuous shaking at 30 ± 1 °C for 24 h. The obtained bacterial cells were used for the fabrication of MR-1@AuNPs by biomineralization. The MR-1 cells were washed with sterile water 3 times and then suspended in an aqueous solution of chloroauric acid ([${\text{AuCl}}_{4}^{-}$] = 1.5 mM liter^−1^, 15 ml). After 16 h of incubation, the color of the solution turned from light yellow to purple-black, suggesting that ${\text{AuCl}}_{4}^{-}$ was converted to AuNPs for the formation of Au-coated MR-1 cells (MR-1@AuNPs) [48].

### Fabrication of the electrochemical cell

The transparent SLG electrode used in this work was constructed according to the method reported in our previous work. A 47-nm-thick gold film was coated on a cover slide, followed by treatment of gold etchant for 1 min in the center. The remaining gold film was used to connect the graphene and the potentiostat. A CVD graphene sample was transferred onto the etched hole of the gold substrate with a polymethyl methacrylate (PMMA)-mediated approach. Simply, a layer of PMMA was spin-coated onto the graphene, and the metal below it was etched away completely. The PMMA/graphene stack was then transferred onto the Au surface. After the graphene was transferred onto the gold substrate, the PMMA layer was dissolved and removed by acetone. To attain anaerobic condition and allow the solution exchange, we construct a sealed incubator on an SLG substrate as described in the previous work [39]. Two concentric PDMS wells are attached and sealed to the substrate, allowing for solution exchange and N_2_ atmosphere. After MR-1 cell immobilization, the electrochemical cell is thoroughly rinsed with the deaerated M9 buffer (containing 18 mM lactate) [11]. To minimize the effect of secreted mediators, electrolyte solution is exchanged with new deaerated M9 buffer (containing 18 mM lactate) after each LSV scan, and the ambient temperature is kept constant at 30 °C for cell culture and detection. The potential of graphene was controlled with respect to Ag/AgCl reference electrode with the potentiostat using a platinum wire as the counter electrode.

### Simultaneous electrochemical and scattering measurements

The electrochemical cell was placed on the 100× oil immersion objective (NA = 1.49) equipped with a Nikon Ti-E inverted microscope. N_2_ flow into the electrochemical cell was started just before the beginning of the measurements. After collecting the cells by centrifugation, they were resuspended in deaerated M9 buffer (containing 18 mM lactate) and further cultured with chronoamperometry at +400 mV versus Ag/AgCl for at least 1 h. LSV was performed for all bacterial cells on the SLG during the growth cycles at maximum current density. Scattering imaging and LSV measurements were started simultaneously. In LSV measurements, the WE potential was swept at 10 mV/s from −0.2 V to 0.6 V, while the frame rate was 500 Hz for all optical measurements.

### Conversion of the single-cell LSV curve

The time-dependent scattering intensity *I* of single MR-1 cell was recorded from the image sequence during the LSV scanning with Fiji software. According to our previous work [38], the current density *i* of a single cell can be simply measured by *i* = *Aed*(∆*I*/*I*)/*dt*, where *e* is the elementary charge, ∆*I*/*I* is the relative scattering change of a single cell, and *A* is a constant that measures the response slope of ∆*I*/*I* to the charge density of graphene [38]. To further convert the current density to the current, we calculate the contact area *S* of the target MR-1 cell and calculate the current by *I* = *Si*. Then, we plot the calculated current to the applied potential, resulting in the single-cell LSV curve.

## Data Availability

The data are available from the authors upon a reasonable request.
