# Supplementary material for: In Vivo Voltammetric Imaging of Metal Nanoparticle-Catalyzed Single-Cell Electron Transfer by Fermi Level-Responsive Graphene
Source: Research (Wash D C). 2023 May 22;6:0145. doi: 10.34133/research.0145 (PMC10200910; doi:10.34133/research.0145)

Supporting Information for

**In-Vivo Voltammetric Imaging of Metal Nanoparticles-Catalyzed Single-Cell Electron Transfer by Fermi Level-Responsive Graphene**

Qing Xia, Rui Liu, Xueqin Chen, Zixuan Chen* and Jun-Jie Zhu*

State Key Laboratory of Analytical Chemistry for Life Science and Collaborative Innovation Center of Chemistry for Life Sciences, School of Chemistry and Chemical Engineering, Nanjing University, Nanjing 210023, PR China.

Email: [chenzixuan@nju.edu.cn](mailto:chenzixuan@nju.edu.cn), [jjzhu@nju.edu.cn](mailto:jjzhu@nju.edu.cn).

**Table of contents**

Supporting Figures S2

**Supporting Figures**

**Figure S1.** (a) Cycle voltammograms of MR-1 cells with (blue) and without (black) cultured in LB containing 10 μM flavin before immobilized on the SLG electrode. The electrolyte is M9 buffer and the scan rate is 0.01 V s^-1^. (b) Differential pulse voltammetry of the carbon cloth working electrode dipped in the MR-1 suspensions in LB after poised a potential of 0.24 V for 0 (blue), 24 (red) and 48 h (green).

**
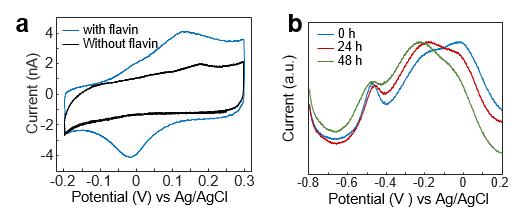
**

**Figure S2.** Snapshots of Video S1 showing the differential scattering images of single MR-1 cells during the LSV scanning. The electrolyte is M9 buffer and the scan rate is 0.01 V s^-1^.

**
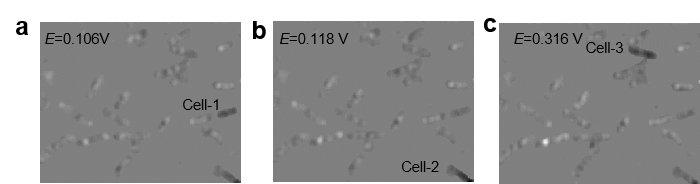
**

**Figure S3.** (a) Scattering image of single gold nanostars (GNS) on the SLG. (b) Scattering light intensity of single GNS and the SLG area during the linear scanning of applied potential.

**
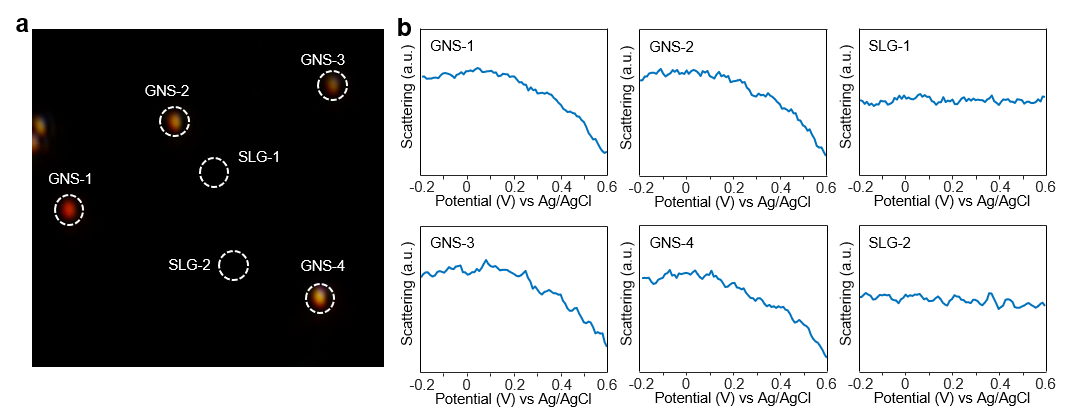
**

**Figure S4.** Converted linear sweep voltammograms of different individual MR-1 cells. The electrolyte is M9 buffer and the scan rate is 0.01 V s^-1^.


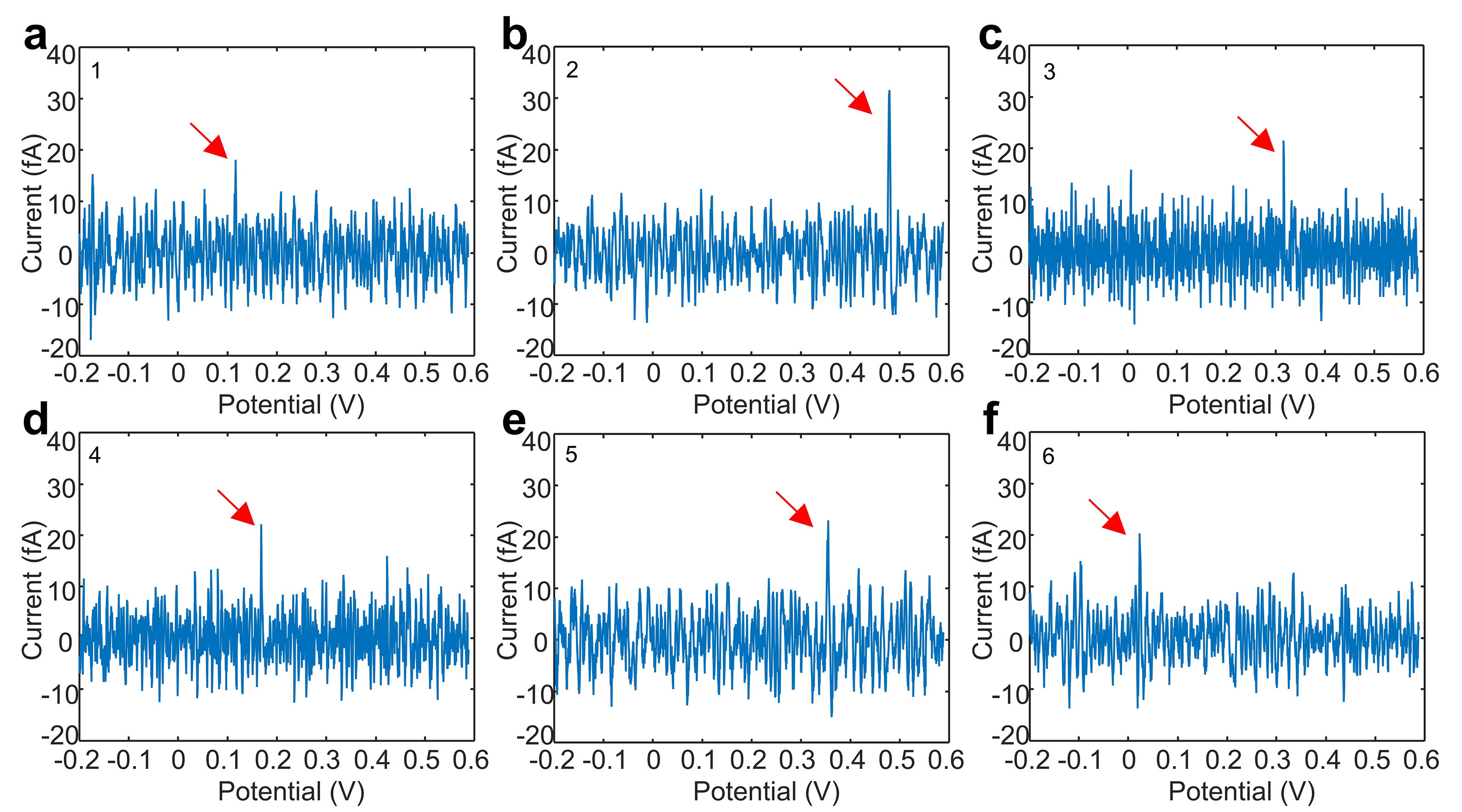


**Figure S5.** Correlativity of scattering intensities vs peak currents with Pearson correlation (R and P-value shown). The shaded region indicates the 95% prediction interval of the fitting. n=51.


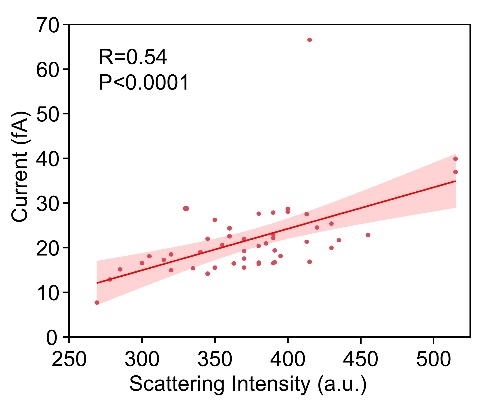


**Figure S6.** Electrochemical analysis of MR-1. (a-b) Nonturnover linear sweep voltammograms of single MR-1 cells in absence of the substrate lactate. The electrolyte is M9 buffer and the scan rate is 0.01 V s^-1^. (c) Statistical turnover (blue, n = 50) and nonturnover (yellow, n = 46) peak current of MR-1 cells. Line, median; Bottom and top of boxes, first and third quartiles, respectively. **** (P < 0.0001) denotes statistically significant difference. (d) Histograms showing the distribution of single oxidation events in nonturnover process. The bulk linear sweep voltammogram (black line) measured with a potentiostat is also shown.


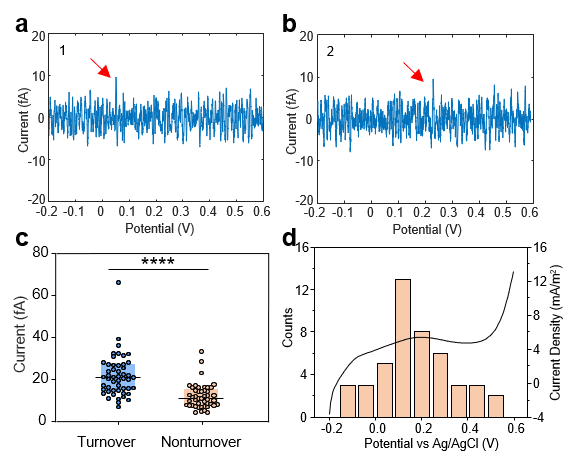


**Figure S7.** (a) Cycle voltammograms of ΔCymA MR-1 mutant cells. The electrolyte is M9 buffer and the scan rate is 0.01 V s^-1^. (b) Converted linear sweep voltammograms of a single ΔCymA mutant cells.


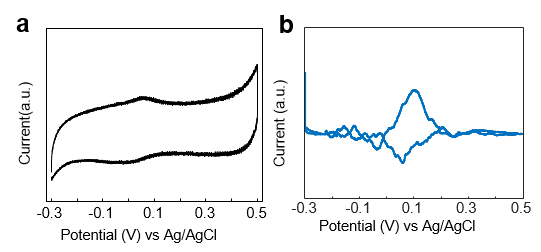


**Figure S8.** (a-b) SEM images of (a) MR-1 cells modified by 35 nm (a) and 14 nm AuNPs(b). The scale bar was 500 nm. Insets showed magnifications of regions marked with dashed boxes. (c-d) Histograms showing the distribution of the size AuNPs on (c) MR-1@Au35 (n=80) and (d) MR-1@Au14 (n=100).


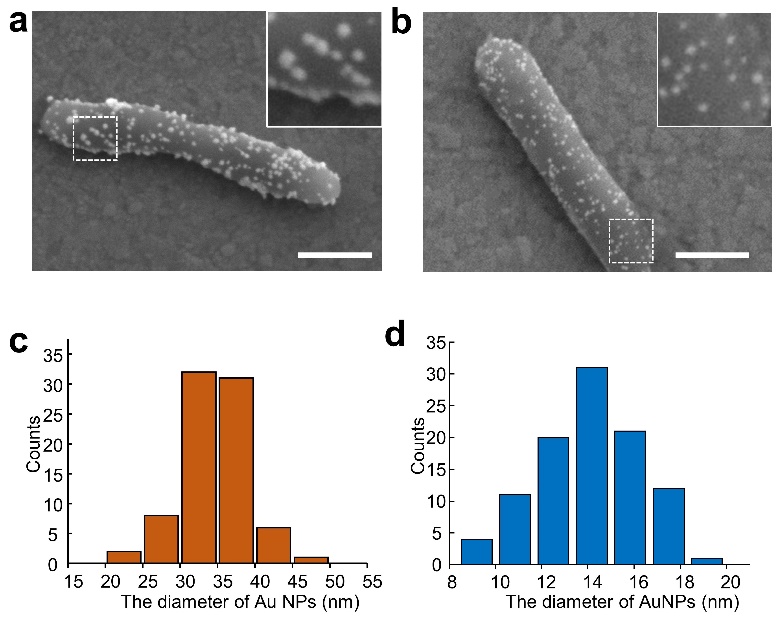


**Figure S9.** Viability analysis of MR-1@AuNPs and MR-1. The bright-field image, SYTO 9 fluorescent image (live cells), Propidium Iodide (PI) fluorescent image (dead cells) and the merged images of (a) MR-1@AuNPs and (b) native MR-1.


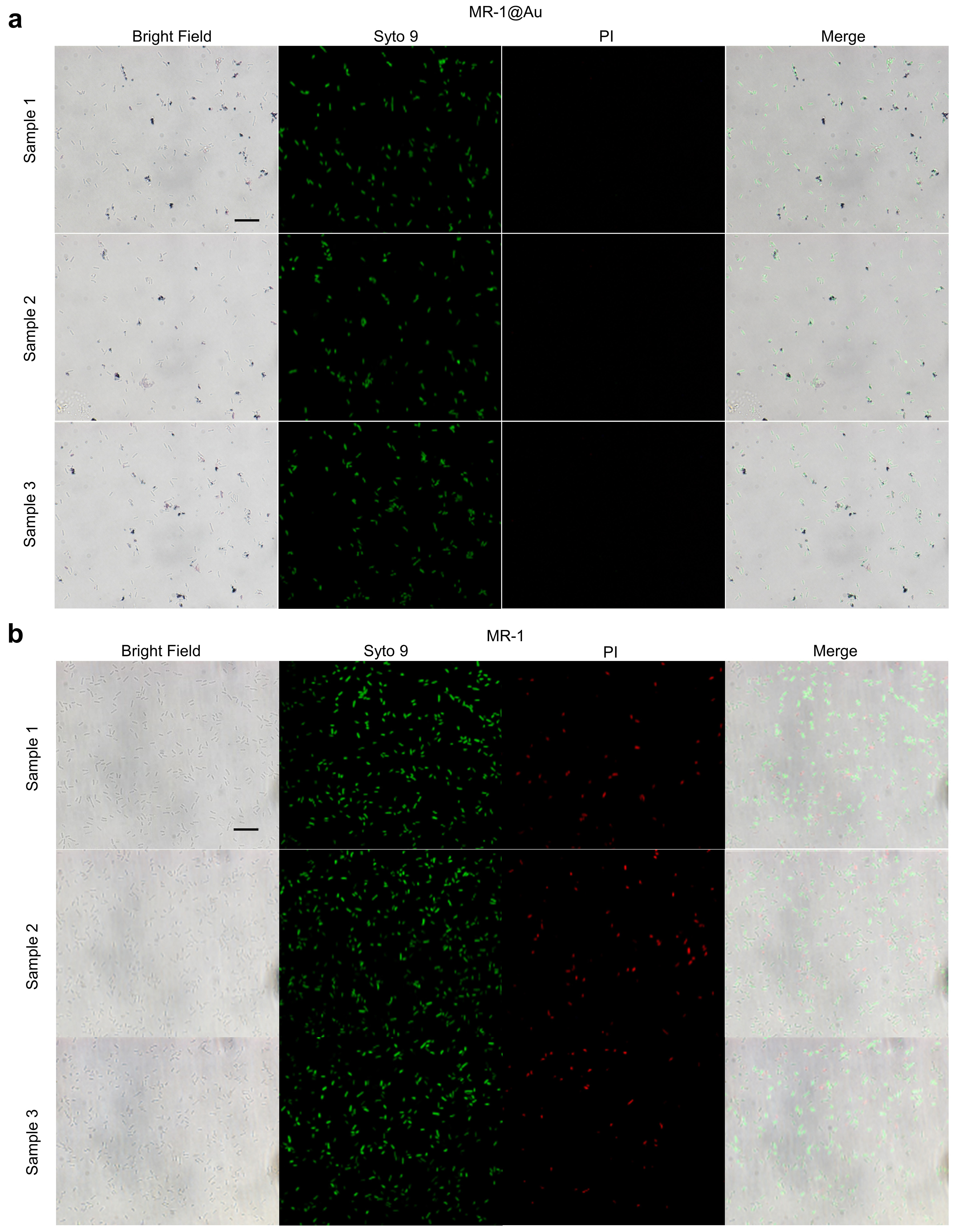


**Figure S10.** Chronoamperometry of monodispersed MR-1@AuNPs cells (a) in the presence of 18 mM lactate and (b) in the absence of lactate on the SLG electrode surface at 0.4 V (vs. Ag/AgCl).


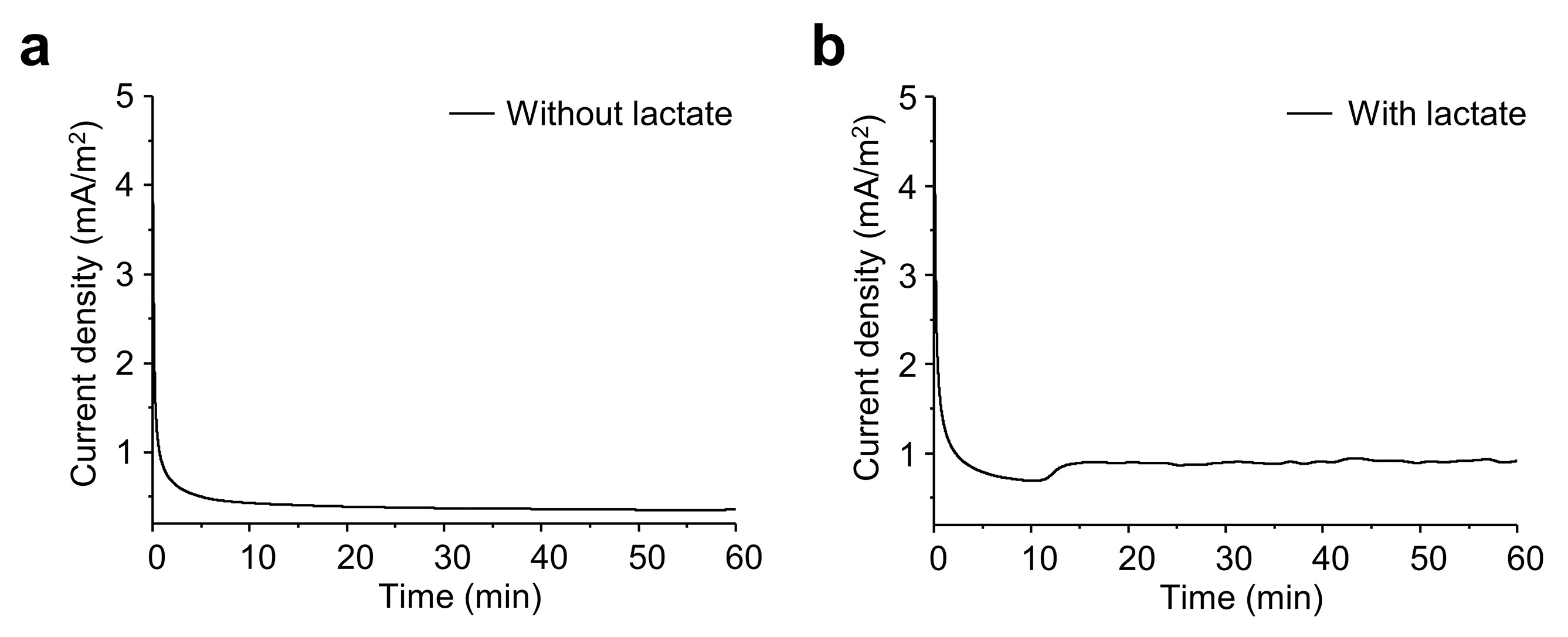


**Figure S11.** Bulk linear sweep voltammograms of MR-1@AuNPs (red) and MR-1 (black) measured with a potentiostat. The electrolyte is M9 buffer and the scan rate is 0.01 V s^-1^.

**
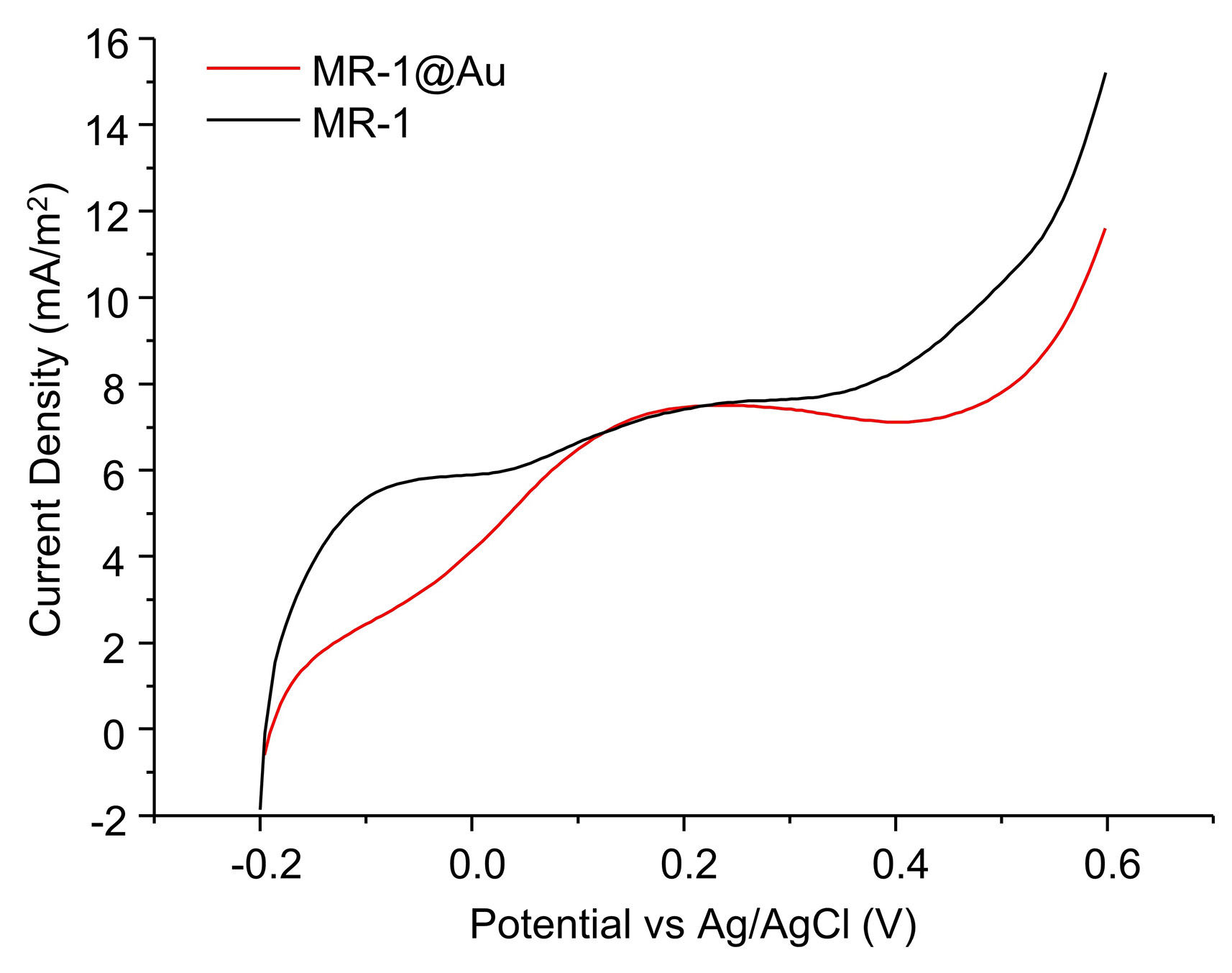
**

**Figure S12.** (a) Linear sweep voltammograms of the whole MR-1 cell-1. (b-c) Linear sweep voltammograms of cell-1 at (b) left part and (c) right part. (d) Linear sweep voltammograms of the whole MR-1 cell-2. (e-f) Linear sweep voltammograms of cell-1 at (e) left part and (f) right part. (g) Linear sweep voltammograms of the whole MR-1 cell-3. (h-i) Linear sweep voltammograms of cell-1 at (h) left part and (i) right part. (j) Linear sweep voltammograms of the whole MR-1 cell-4. (k-l) Linear sweep voltammograms of cell-1 at (k) left part and (l) right part.


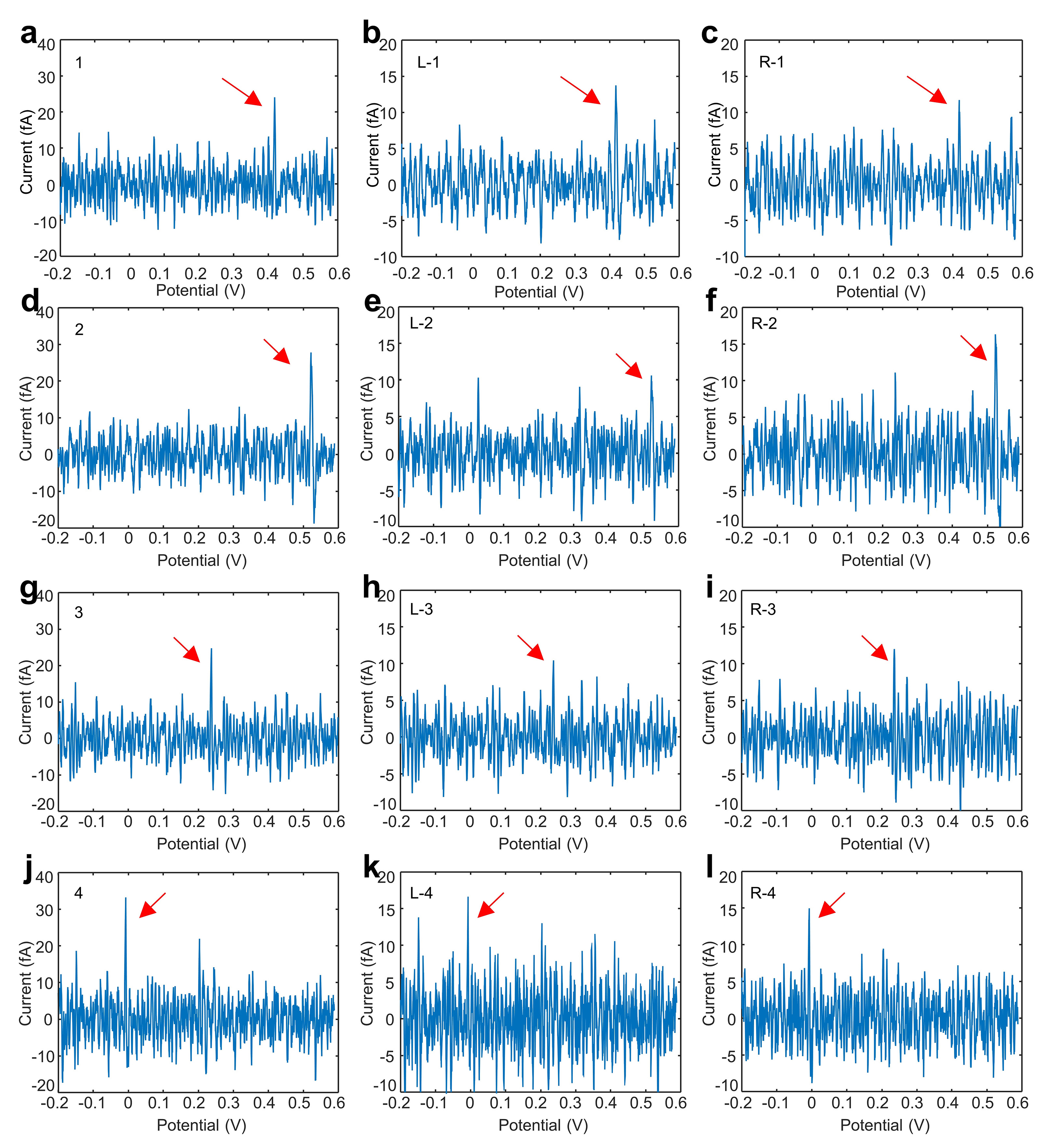


**Figure S13.** Linear sweep voltammograms of the whole (a) Cell-1, (b) Cell-2 and (c) Cell-3 in Figure 5.


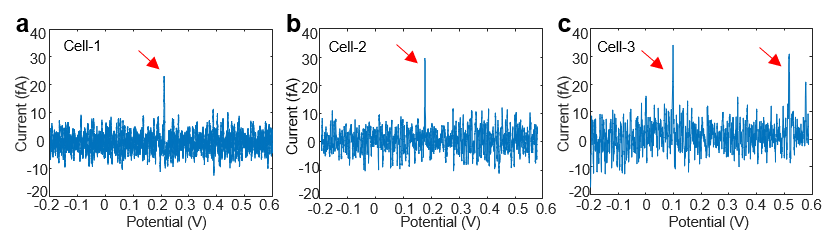


**Figure S14.** Nonturnover electrochemical analysis of MR-1@AuNPs. (a-d) Nonturnover linear sweep voltammograms of MR-1@AuNPs. The electrolyte is M9 buffer and the scan rate is 0.01 V s^-1^. Insets showing the scattering images (4 by 4 μm) of the MR-1@AuNPs cells. (e) Histograms showing the distribution of single oxidation events in nonturnover (blue, n = 35) process. The bulk linear sweep voltammograms (black line) measured with a potentiostat are also shown. (f) Histograms showing the distribution of oxidation current in nonturnover (blue, n = 35) process.


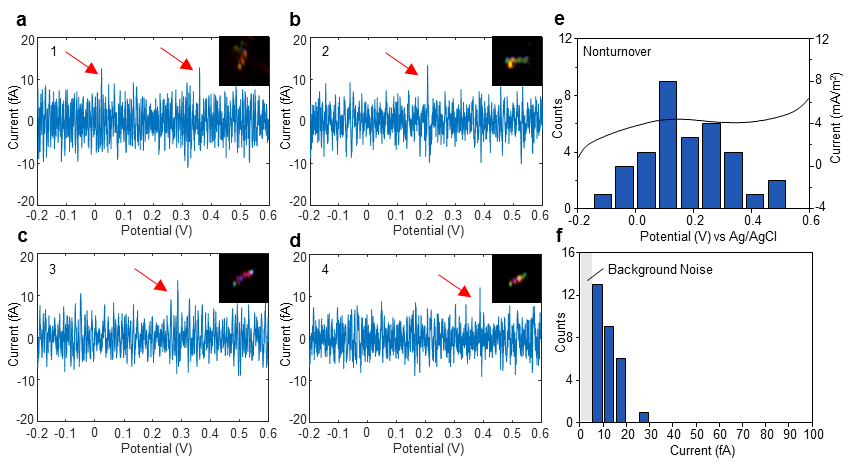

Supplement: Supplementary 1 — Figs. S1 to S14 Video S1 [file research.0145.f1.zip › SupportingInformation.docx]
